# Supplementary material for: Using COVID-19 pandemic perturbation to model RSV-hMPV interactions and potential implications under RSV interventions
Source: Nat Commun. 2025 Aug 6;16:7261. doi: 10.1038/s41467-025-62358-w (PMC12328645; doi:10.1038/s41467-025-62358-w)
Supplement: Supplementary file 1 — Supplementary Information [file 41467_2025_62358_MOESM1_ESM.pdf]

Supplementary Information for *Using COVID-19 pandemic perturbation to model RSV-hMPV interactions and potential implications under RSV interventions*

1 Discretized model

We discretized the system of differential equations presented in the main text following He *et al.* [1], using 1 week time steps. For each pathogen, $i \in \{\text{RSV}, \text{hMPV}\}$, we define the number of individuals that leave each compartment at time t, as

$$\Delta S_i[t] = (1 - \exp(-\lambda_i - \mu))S_i[t-1] \quad (1)$$
$$\Delta E_i[t] = (1 - \exp(-\sigma - \mu))E_i[t-1] \quad (2)$$
$$\Delta I_i[t] = (1 - \exp(-\gamma - \mu))I_i[t-1] \quad (3)$$
$$\Delta R_i[t] = (1 - \exp(-\omega - \mu))R_i[i-1] \quad (4)$$

where λ_i is the force of infection for pathogen i (defined in Methods), σ is the rate at which exposed individuals become infectious, γ is the rate at which infected individuals recover, ω is the rate at which recovered individuals lose their immunity, and μ is the birth and death rate. The total number of individuals leaving each compartment contains those that are changing disease state (e.g., newly infected individuals move from S to E) as well individuals leaving the population due to death. We then define the number of individuals transitioning between compartments (and not dying) by the rules of competing rates [2].

$$N_{S_i \to E_i}[t] = \Delta S_i[t](\lambda_i/(\mu + \lambda_i)) \quad (5)$$
$$N_{E_i \to I_i}[t] = \Delta E_i[t](\sigma/(\mu + \sigma)) \quad (6)$$
$$N_{I_i \to R_i}[t] = \Delta I_i[t](\gamma/(\mu + \gamma)) \quad (7)$$
$$N_{R_i \to S_i}[t] = \Delta R_i[t](\omega/(\mu + \omega)) \quad (8)$$

Then, we can define the discretized model as follows:

$$S_i[t] = S_i[t-1] + \mu N + N_{R_i \to S_i}[t] - \Delta S_i[t] \quad (9)$$
$$E_i[t] = E_i[t-1] + N_{S_i \to E_i}[t] - \Delta E_i[t] \quad (10)$$
$$I_i[t] = I_i[t-1] + N_{E_i \to I_i}[t] - \Delta I_i[t] \quad (11)$$
$$R_i[t] = R_i[t-1] + N_{I_i \to R_i}[t] - \Delta R_i[t] \quad (12)$$

We follow the same discretization scheme for the SIR model and the independent SEIR models.

2 Model extensions

2.1 Age structure

Age structure is known to be an important component of RSV and hMPV dynamics. As a first pass at this problem, we incorporated age-structure into the population-level model described in the main text. Because fitting a fully age-structured model requires detailed age-specific data and assumptions, we instead rescaled parameter estimates to accommodate heterogeneous mixing assumptions and generate equivalent aggregate outbreak dynamics. Here we describe our approach.

First, we redefined the simple homogeneous mixing model to include a discrete set of age classes $a \in \mathbf{A}$:

$$\frac{dS_{i,a}}{dt} = \mu(N - S_{i,a}(t)) - \lambda_{i,a} S_i(t) + \omega R_{i,a}(t) \tag{13}$$

$$\frac{dE_{i,a}}{dt} = \lambda_{i,a} S_{i,a}(t) - \sigma E_{i,a}(t) - \mu E_{i,a}(t) \tag{14}$$

$$\frac{dI_{i,a}}{dt} = \sigma E_{i,a}(t) - \gamma I_{i,a}(t) - \mu I_{i,a}(t) \tag{15}$$

$$\frac{dR_{i,a}}{dt} = \gamma I_{i,a}(t) - \omega R_{i,a}(t) - \mu R_{i,a}(t) \tag{16}$$

We defined the force of infection for RSV and hMPV as

$$\lambda_{\text{RSV},a}(t) = \beta \xi(t) \frac{1}{N} \sum_{\forall b \in \mathbf{A}} \theta_{a,b} I_{\text{RSV},b}(t) \tag{17}$$

$$\lambda_{\text{hMPV}}(t) = \beta r \xi(t) \frac{1}{N} \Big(1 + c \frac{I_{\text{RSV},a}}{N_{RSV,a}}\Big) \sum_{\forall b \in \mathbf{A}} \theta_{a,b} I_{\text{hMPV},b}(t) \tag{18}$$

where N is the total population size, N_a is the population of age a, $\theta_{a,b}$ is a contact matrix defining the number of contacts between individuals of age a and b, and $\xi(t) = (a\cos(2\pi(w(t)/52 - p)) + 1)$ is the seasonal forcing of the transmission rate, which is assumed to be the same for all ages.

Because we estimated parameters using the model with homogeneous mixing assumptions, we had to calculate a correction factor for the baseline transmission rate, β in the age-structured model. We first show how to derive this calculation using the force of infection for RSV. The total number of new infections in the population at time t can be rewritten in terms of total susceptible and infected individuals in the population,

$$\sum_{\forall a \in \mathbf{A}} \lambda_{\text{RSV},a}(t) S_{\text{RSV},a}(t) = \sum_{\forall a \in \mathbf{A}} \beta \xi(t) \frac{1}{N} S_{\text{RSV},a}(t) \sum_{\forall b \in \mathbf{A}} \theta_{a,b} I_{\text{RSV},b}(t) \tag{19}$$

$$= \beta k \xi(t) \frac{1}{N} S_{\text{RSV}}(t) I_{\text{RSV}}(t) \tag{20}$$

using the constant

$$k = \frac{1}{S_{\text{RSV}}(t) I_{\text{RSV}}(t)} \sum_{\forall a \in \mathbf{A}} \sum_{\forall b \in \mathbf{A}} \theta_{a,b} I_{\text{RSV},b}(t) S_{\text{RSV},a}(t)$$

as a correction factor. The same calculation can be derived to adjust the transmission rate of hMPV; the interaction term $\left(1 + c' \frac{I_{\text{RSV},a}}{N_{RSV,a}}\right)$ is not included as it should not be adjusted. We also convert the fixed rates from exposed to infected, for recovery, for waning from discrete to continuous time, where the continuous time rate $r_c = 1 - \exp(-r_d)$.

Using this model, we considered constant, homogeneous mixing assumptions (as in the unstructured model of the main text) as well as a model that includes heterogeneous mixing patterns as

described by the POLYMOD study [3]. We used 1 month age classes for the first 5 years of life, then 1 year age classes from age 6 until age 10, and then used 5 year age classes through age 70. The final age class includes all individuals over 70 years of age. In this model, incidence is the same when summed across the entire population (as constructed by the correction factor), but the age-specific incidence patterns vary (Supplementary Figure 10).

2.2 Interactions based on lagged incidence

We also considered the possibility that prior (rather than current) RSV infections could affect current hMPV incidence (e.g., through innate or adaptive immunity). We adapted the model presented in the main text to include these potential lags and refit the data from Scotland. Define a lag, l, which is the number of prior weeks of RSV infections that affect current hMPV force of infection. If $l = 0$, only current RSV incidence is used. Then define hMPV force of infection as

$$\lambda_{\text{hMPV}} = \beta r \xi(t) \Big(1 + c \frac{\sum_{i=0}^{l} I_{\text{RSV}}(t-i)}{N}\Big) \frac{I_{\text{hMPV}}(t)}{N}.$$

One limitation of this formulation is the potential for double counting individuals that have been reinfected over the lagged period (enabled by waning immunity). For shorter lags, this is unlikely in practice, and the proportion of individuals in the model for which this will occur is small. Thus, we focus on these short lags only (here, $l = 1, 2, ..., 7$). For each of these lags, we refit the model and compared parameter estimates(Supplementary Figure 11, 12), fits (Supplementary Figure 13), and model performance (Supplementary Figure 14).

3 Supplementary Tables

Supplementary Table 1: List of parameter assumptions and priors.

Parameter	Value or Prior	Source
incubation rate	$\sigma = 7$ weeks^{-1}	Reis & Shaman [4]*
recovery rate	$\gamma = 1.167$ weeks^{-1}	Reis & Shaman [4]*
waning rate	$\omega = 0.0098$ weeks^{-1}	White *et al.* [5]
Scotland population size	$N = 5.436$ million	NRS† 2022 census [6]
birth and death rate	$\mu = 0.0002$ weeks^{-1}	NRS† [7]
baseline transmission rate of RSV	$\beta_{\text{RSV}} \sim \text{Norm}(3, 1)$	Reis & Shaman [4]
relative transmission rate of HMPV	$r \sim \text{Norm}(1, 0.2)$	uninformative prior
phase of seasonal forcing	$p \sim \text{Norm}(0.5, 0.1)$	uninformative prior
amplitude of seasonal forcing	$a \sim \text{Norm}(0.5, 0.1)$	uninformative prior
effect of RSV on hMPV transmission rate	$c \sim \text{Norm}(0, 0.2)$	uninformative prior
reporting rate for RSV	$\rho_{\text{RSV}} \sim \text{Beta}(1, 99)$	uninformative prior
reporting rate for HMPV	$\rho_{\text{HMPV}} \sim \text{Beta}(1, 99)$	uninformative prior
RSV initial susceptible proportion	$S_{\text{RSV}}[1]/N \sim \text{Beta}(2, 98)$	uninformative prior‡
RSV initial exposed proportion	$E_{\text{RSV}}[1]/N \sim \text{Beta}(2, 98)$	uninformative prior‡
RSV initial infected proportion	$I_{\text{RSV}}[1]/N \sim \text{Beta}(2, 98)$	uninformative prior‡
HMPV initial susceptible proportion	$S_{\text{HMPV}}[1]/N \sim \text{Beta}(2, 98)$	uninformative prior‡
HMPV initial exposed proportion	$E_{\text{HMPV}}[1]/N \sim \text{Beta}(2, 98)$	uninformative prior‡
HMPV initial infected proportion	$I_{\text{HMPV}}[1]/N \sim \text{Beta}(2, 98)$	uninformative prior‡
RSV negative binomial shape parameter	$\phi_{\text{RSV}} \sim \text{Norm}(0, 10)$	uninformative prior
HMPV negative binomial shape parameter	$\phi_{\text{HMPV}} \sim \text{Norm}(0, 10)$	uninformative prior

* Approximately 1 week infectious period was taken from Reis & Shaman [4], and for the SEIR model, the 1 week infectious period was separated into a 1 day incubation period and 6 day infectious period

† NRS = National Records of Scotland; birth rate calculated directly from data

‡ Priors for initial conditions were specified as a percent of the population, such that the initial percent of the population in the recovered class for pathogen i is $R_i[1] = 1 - (S_i[1] + E_i[1] + I_i[1])$. The values for $S_i[1]$,$E_i[1]$, $I_i[1]$ were chosen to be small to reflect high population-level seroprevalence [8]. Sampling of MCMC starting value was constrained to Beta $\sim (20, 80)$ to prevent the algorithm from initializing out of the $[0, 1]$ range while retaining variability.

Supplementary Table 2: List of parameter estimates from SEIRS model with interaction, including median estimate and 90% credible interval in parenthesis.

**Parameter**	**Estimate (90% credible interval)**
baseline transmission rate of RSV (β_{RSV})	4.10 (4.05 – 4.15)
baseline transmission rate of HMPV ($\beta_{\text{HMPV}} = r\beta_{\text{RSV}}$)	3.40 (3.25 – 3.58)
phase of seasonal forcing (p)	0.52 (0.51 – 0.53)
amplitude of seasonal forcing (a)	0.23 (0.22 – 0.24)
effect of RSV on hMPV transmission rate (c)	-0.39 (-0.43 – -0.34)
reporting rate for RSV (ρ_{RSV})	0.0012 (0.0012 – 0.0013)
reporting rate for HMPV (ρ_{HMPV})	0.0004 (0.0004 – 0.0005)
RSV initial susceptible proportion ($S_{\text{RSV}}[1]$)	0.2 (0.18 – 0.21)
RSV initial exposed proportion ($E_{\text{RSV}}[1]$)	0.004 (0.001 – 0.007)
RSV initial infected proportion ($I_{\text{RSV}}[1]$)	0.002 (0.0005 – 0.005)
HMPV initial susceptible proportion ($S_{\text{HMPV}}[1]$)	0.23 (0.21 – 0.25)
HMPV initial exposed proportion ($E_{\text{HMPV}}[1]$)	0.005 (0.002 – 0.009)
HMPV initial infected proportion ($I_{\text{HMPV}}[1]$)	0.009 (0.005 – 0.014)

4 Supplementary Figures

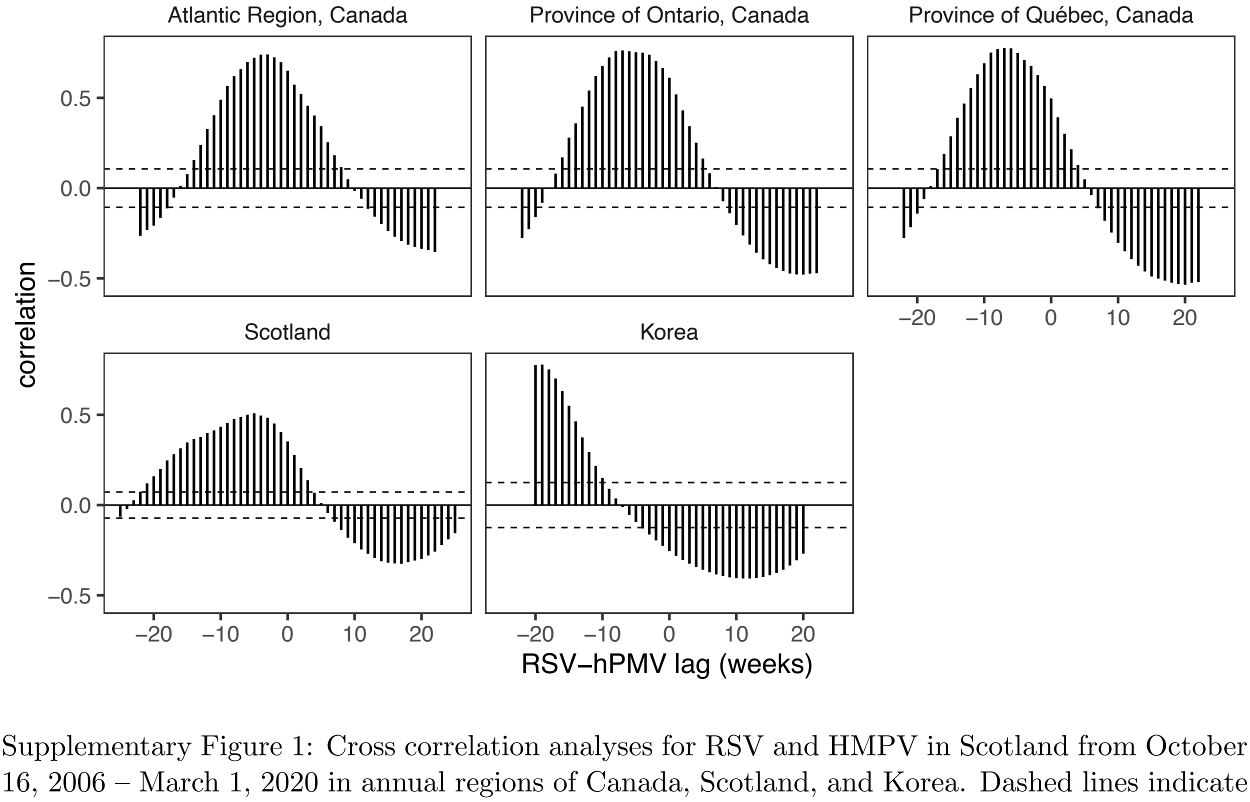

Supplementary Figure 1: Cross correlation analyses for RSV and HMPV in Scotland from October 16, 2006 – March 1, 2020 in annual regions of Canada, Scotland, and Korea. Dashed lines indicate 95% confidence intervals.

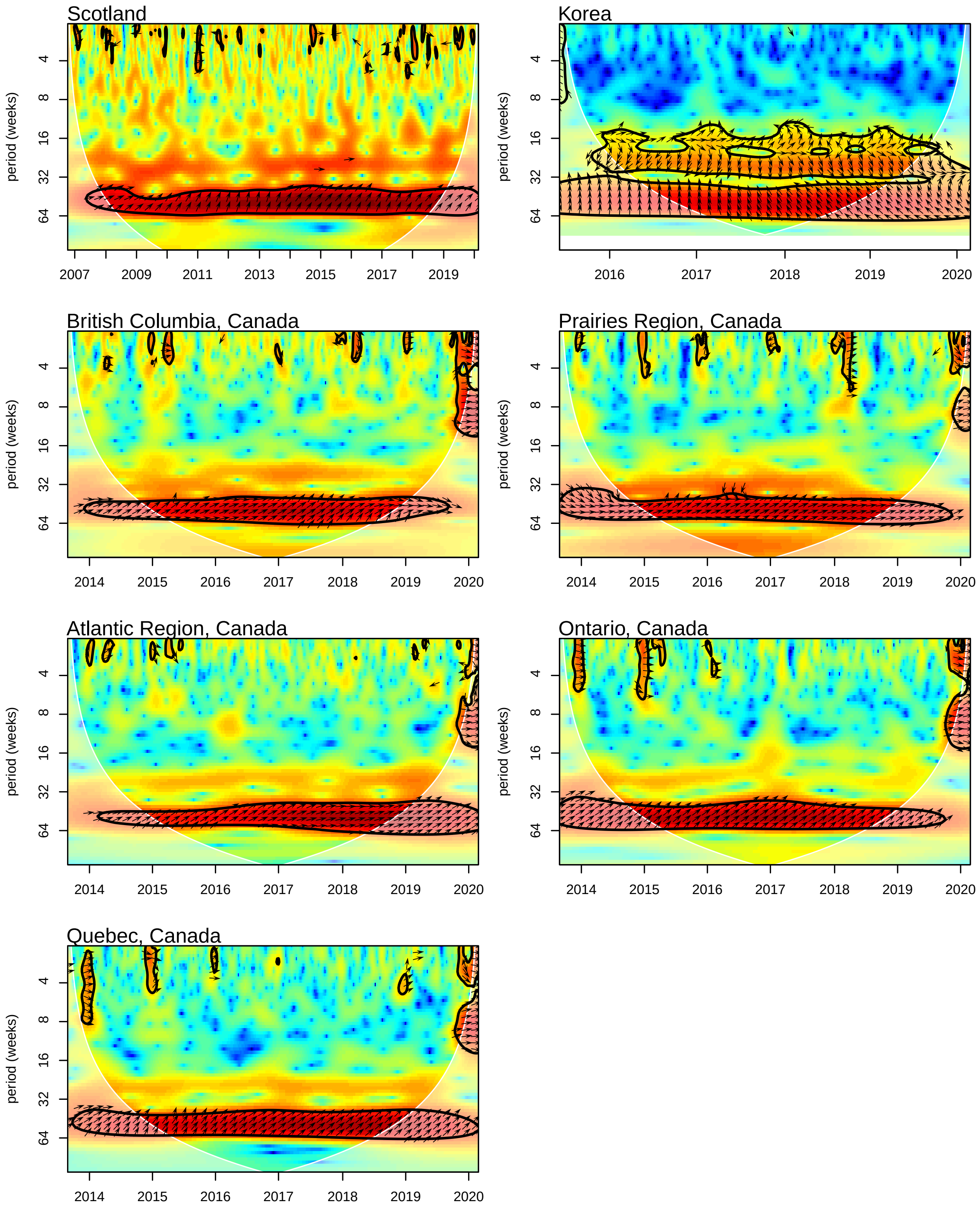

Supplementary Figure 2: Cross wavelet transform of RSV and hMPV detections in each location. Color shows significance and significant regions (95% level) are highlighted with a contour. For significant periods and weeks, arrows indicate the relative phase between RSV and hMPV detections. Arrows pointing right suggest RSV and hMPV detections are in phase (versus out-of-phase for arrows pointing left). The vertical position of the arrows indicate the lags between the two time series. Horizontal arrows suggest no lag between detections. Arrows above horizontal suggest RSV detections lead hMPV detections (where a vertical, upward arrow indicates leading by $\pi/2$). Arrows below horizontal suggest hMPV detections lead RSV (again, vertical, downward arrow indicates leading by $\pi/2$).

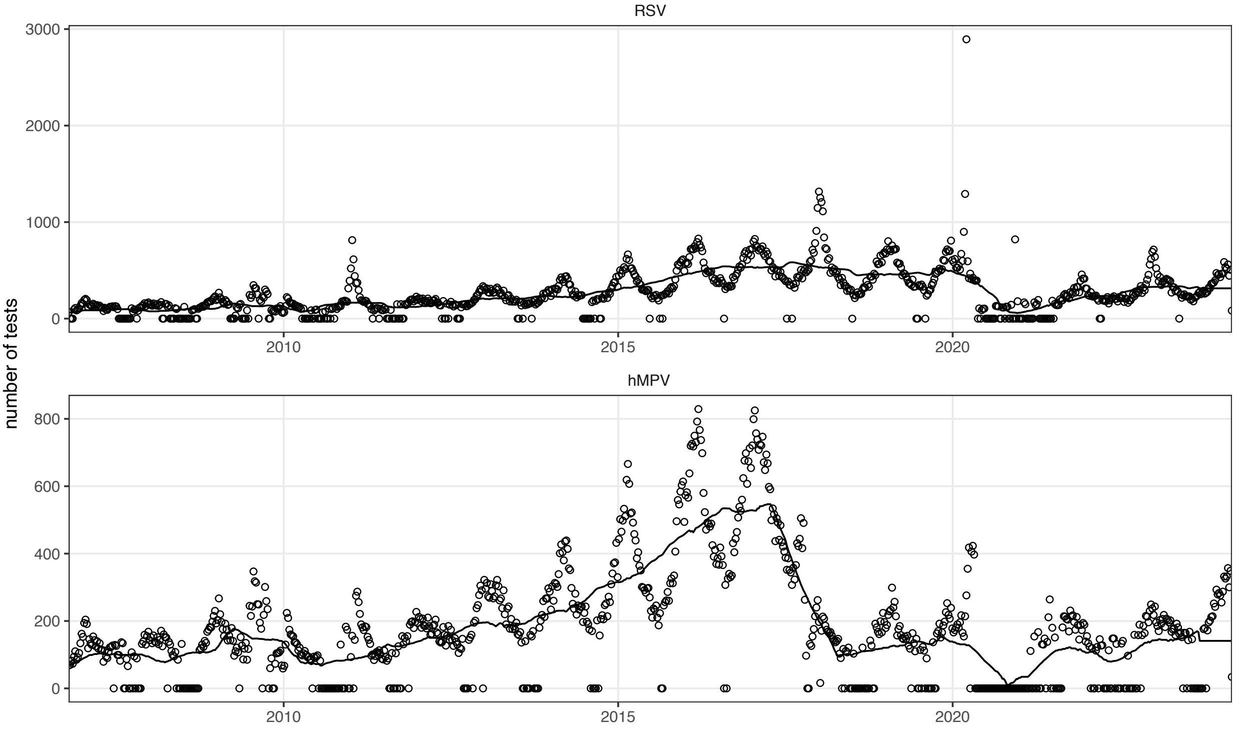

Supplementary Figure 3: Testing trends of RSV (top) and HMPV (bottom) from week of October 16, 2006, through week of March 4, 2024. Points show number of tests performed and black line shows 1 year moving average, which is scaled from 0 to 1 when used as an input in the model.

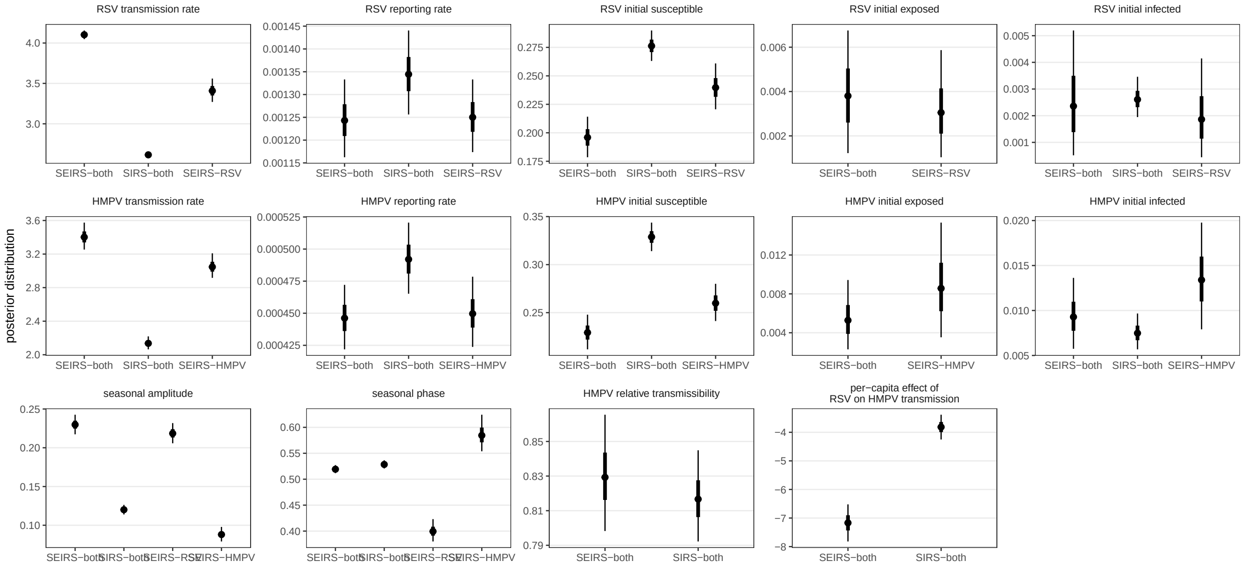

Supplementary Figure 4: Parameter estimates from coupled SEIRS model (SEIRS-both), coupled SIRS model (SIRS-both), and independent SIERS fits (SEIRS-RSV and SEIRS-HMPV). Circle shows median estimate from posterior distribution of $n = 7500$, thick lines show 50% credible interval, and thin lines show 90% credible interval. When a parameter was not estimated for a particular model, it is not shown in the panel (e.g., the initial exposed population is not estimate for the SIR model that does not have an exposed compartment).

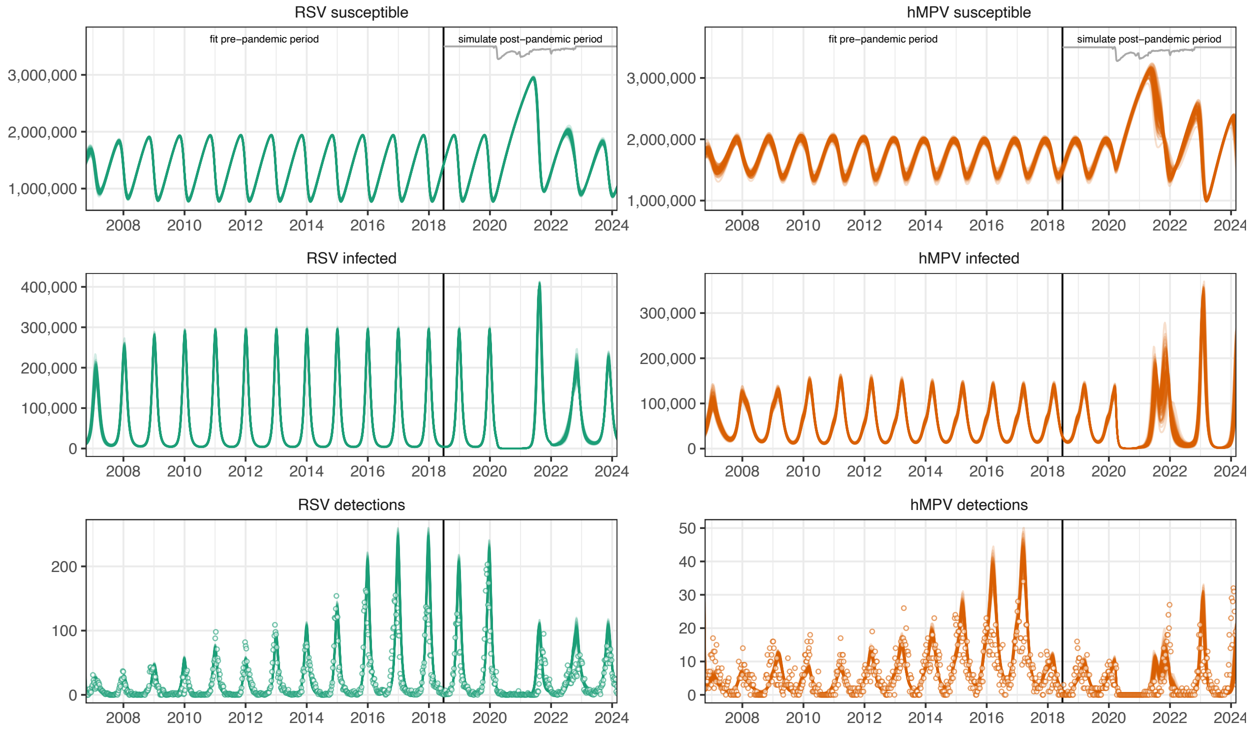

Supplementary Figure 5: Results for coupled SIRS model, which assumes an interaction between RSV (green) and HMPV (orange).Lines show 100 randomly selected model simulations, open circles show observations, and black horizontal line shows date on which fitting period ends and out-of-sample simulations begin. See caption of Figure 3 for details.

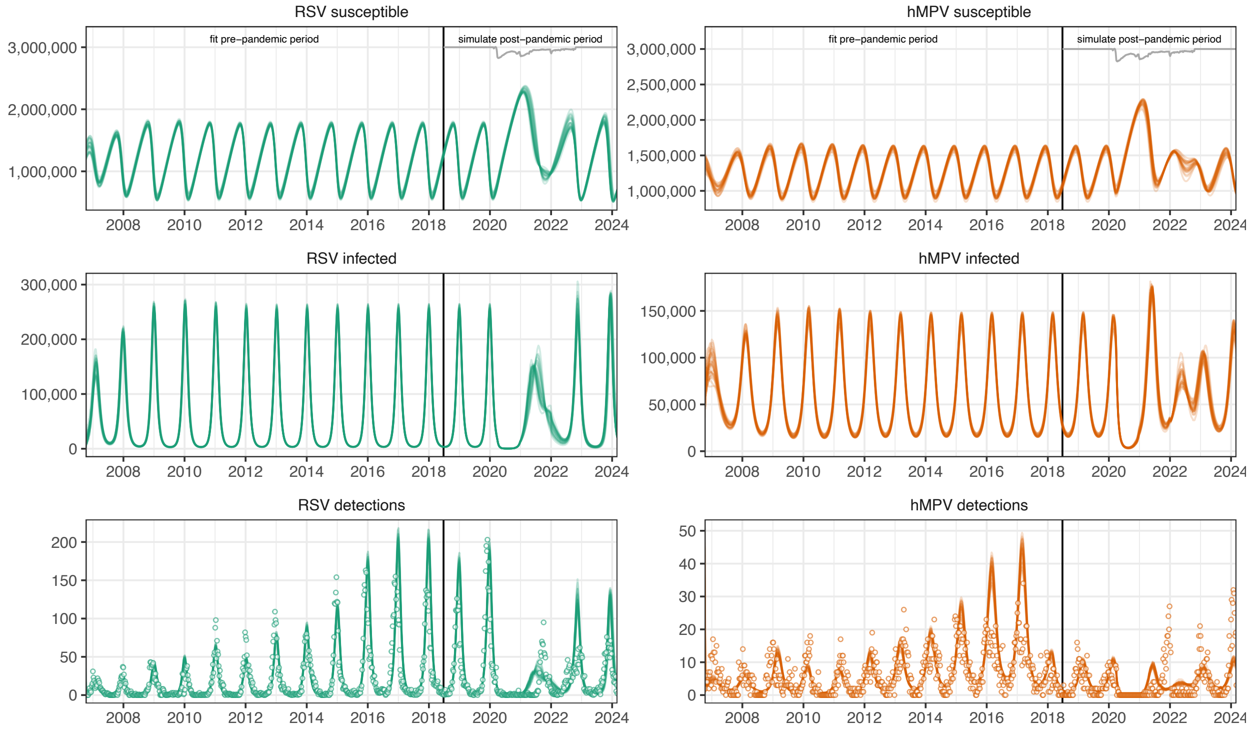

Supplementary Figure 6: Results for independent SEIRS model, which assumes no interaction between RSV (green) and HMPV (orange). Lines show 100 randomly selected model simulations, open circles show observations, and black horizontal line shows date on which fitting period ends and out-of-sample simulations begin. See caption of Figure 3 for details.

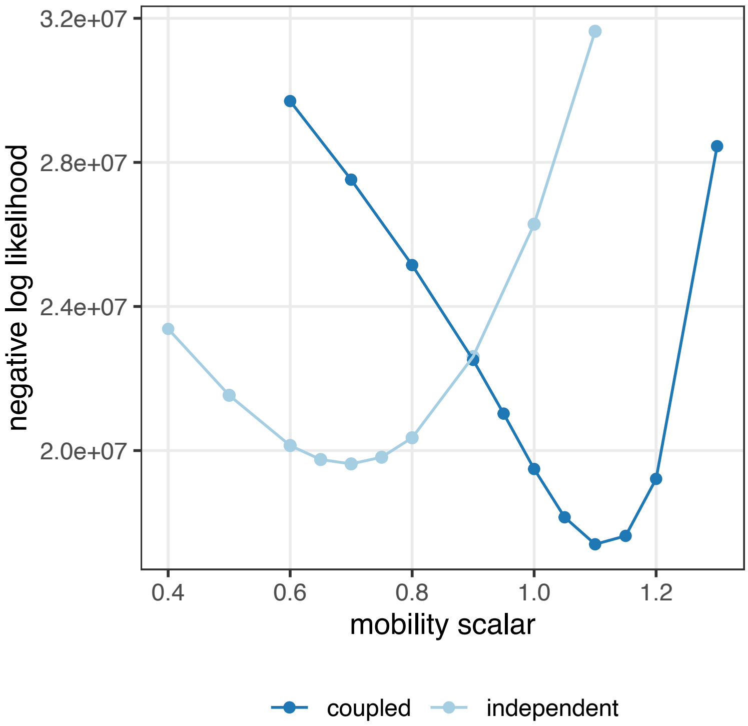

Supplementary Figure 7: Negative log likelihood of out-of-sample model fits for the coupled SEIRS model (dark blue) and the independent SEIRS model (light blue). Coupled model estimates interaction between RSV and HMPV, whereas the independent model assumes no interaction. The mobility scalar (x-axis) indicates the scaling of google mobility changes that correspond to the percent reduction in transmission.

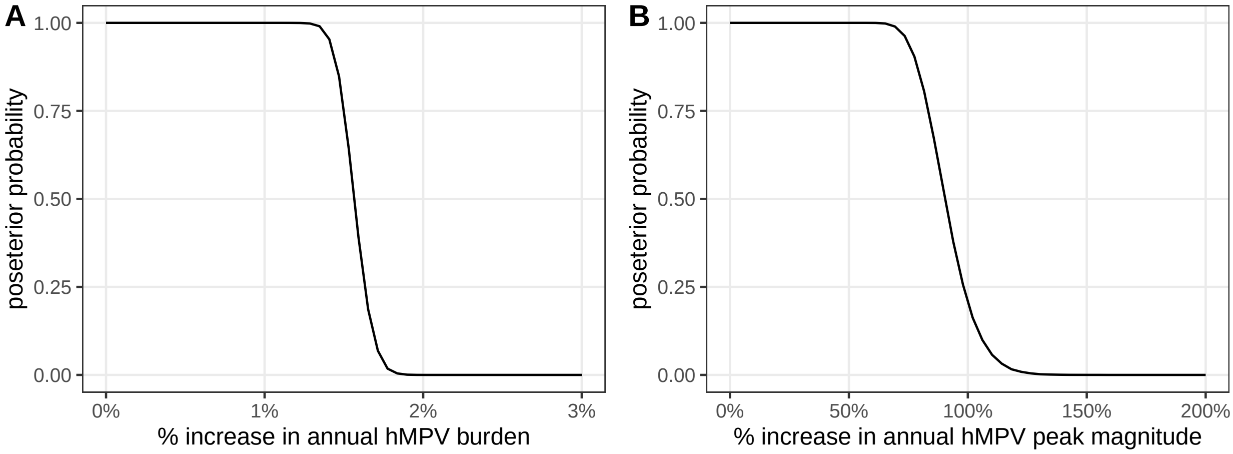

Supplementary Figure 8: Posterior probability of range of percent increases in (A) annual hMPV burden and (B) annual hMPV peak magnitude. The model was simulated to equilibrium for each posterior draw, with and without an RSV interaction. The percent increase in annual burden and peak magnitude were calculated for each posterior simulation. For reference, the average percent increase in annual burden for hMPV outbreaks to be as large as pre-intervention RSV outbreaks is almost 200% for annual burden and 500% for peak burden.

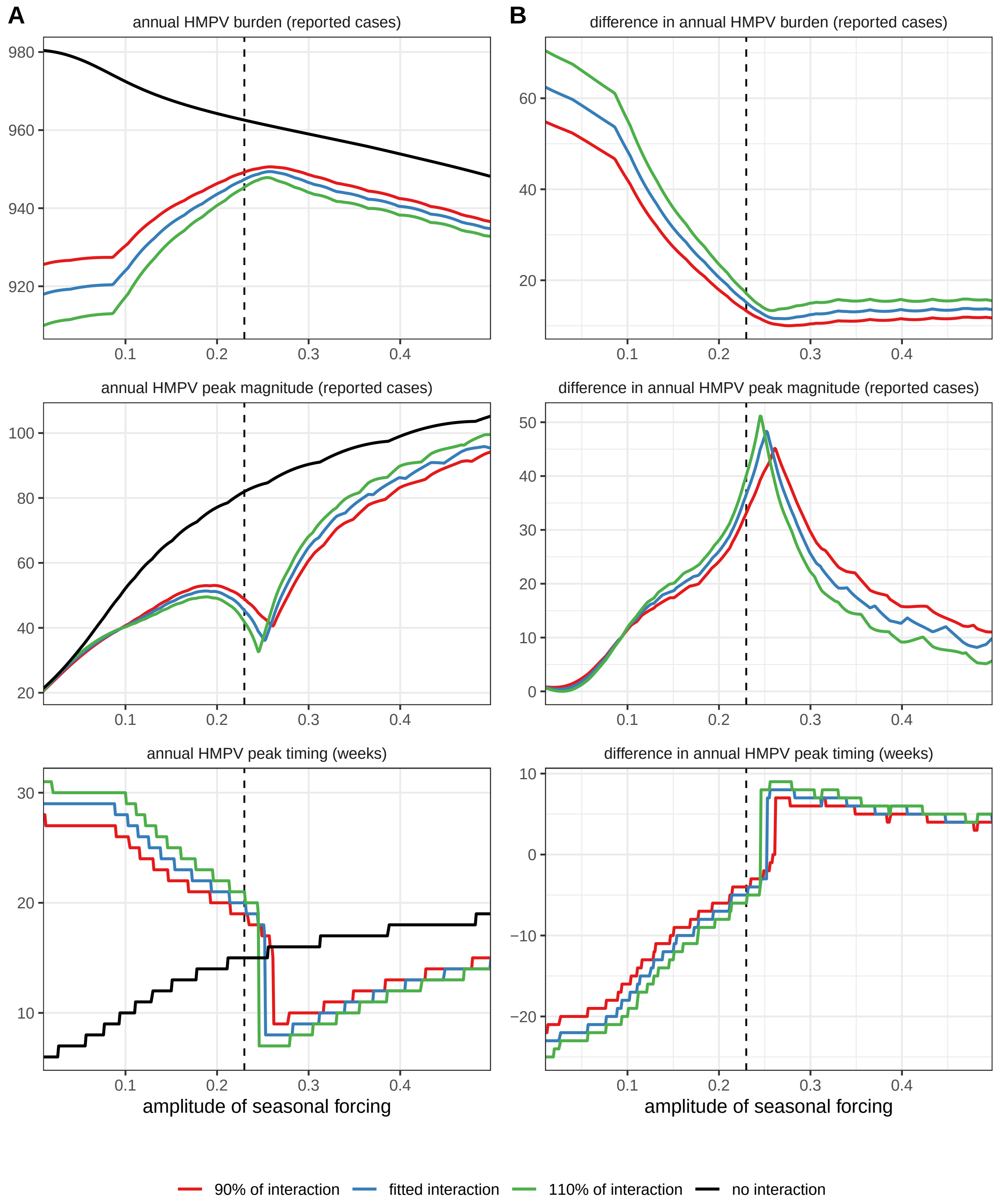

Supplementary Figure 9: Change in annual HMPV burden, peak magnitude, and peak timing as a function of seasonal forcing (x-axis) and effect of RSV infection on HMPV transmission rate including the fitted effect (blue, $c = -7.13$) a 10% stronger effect (green, $c = -7.84$) and a 10% weaker effect (red, $c = -6.42$). Outcomes are compared to a case with no interaction (black). Dashed vertical lines shows the amplitude of seasonal forcing estimated for Scotland.

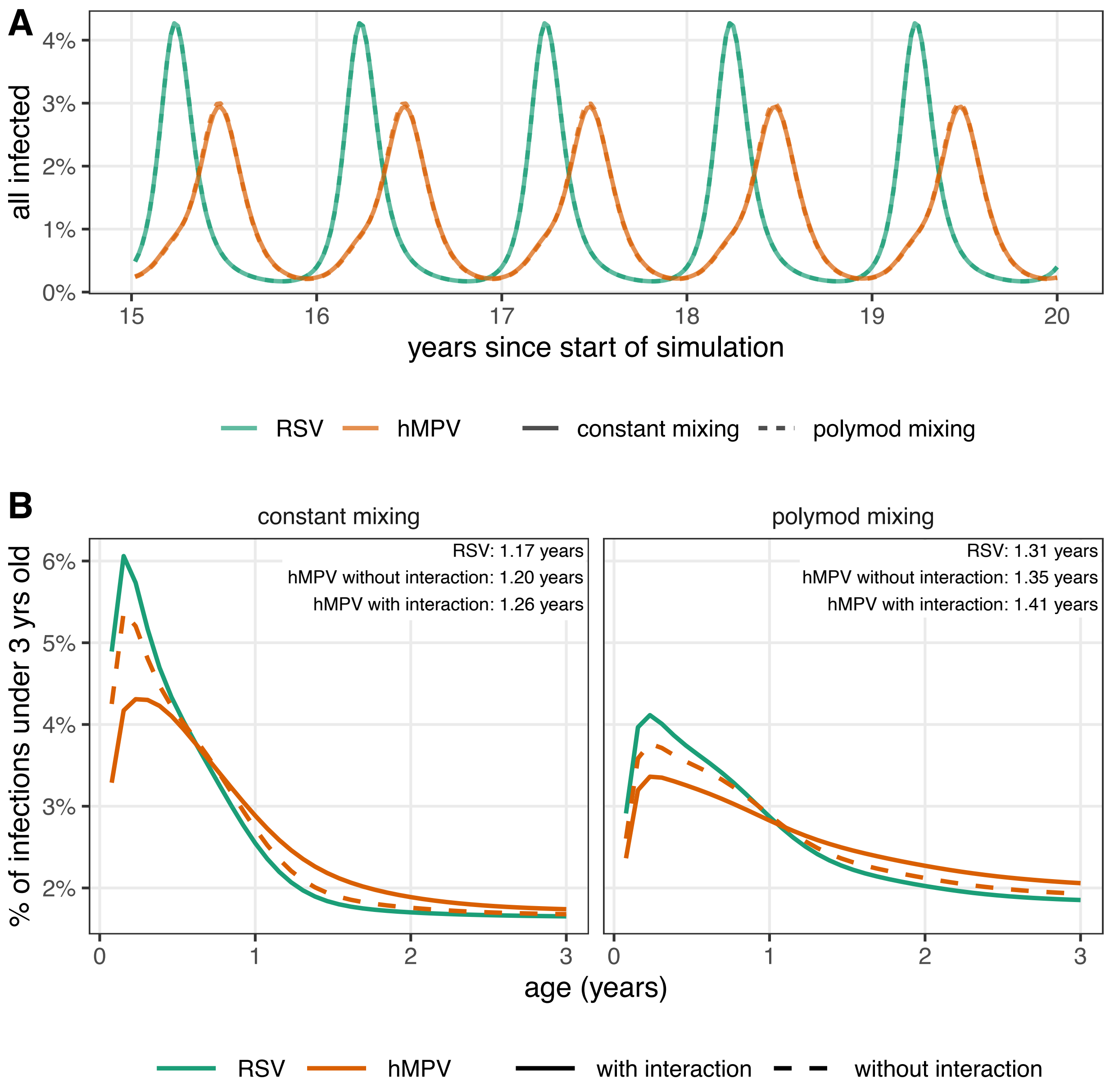

Supplementary Figure 10: Incorporating age structure into RSV-hMPV model. (A) Equilibrium dynamics from age-structured model with constant, homogeneous mixing assumptions (the same as model presented in main text) and with mixing assumptions following the POLYMOD study. (B) Age of infection at equilibrium in the first 3 years of life for constant mixing and POLYMOD mixing assumptions. In each case, the mean age of infection under 3 years is recorded for RSV (green), hMPV without an interaction with RSV (i.e., $c = 0$, orange dashed) and with an interaction with RSV (i.e., $c = -7.13$, orange solid). Later infections are not considered because of waning immunity.

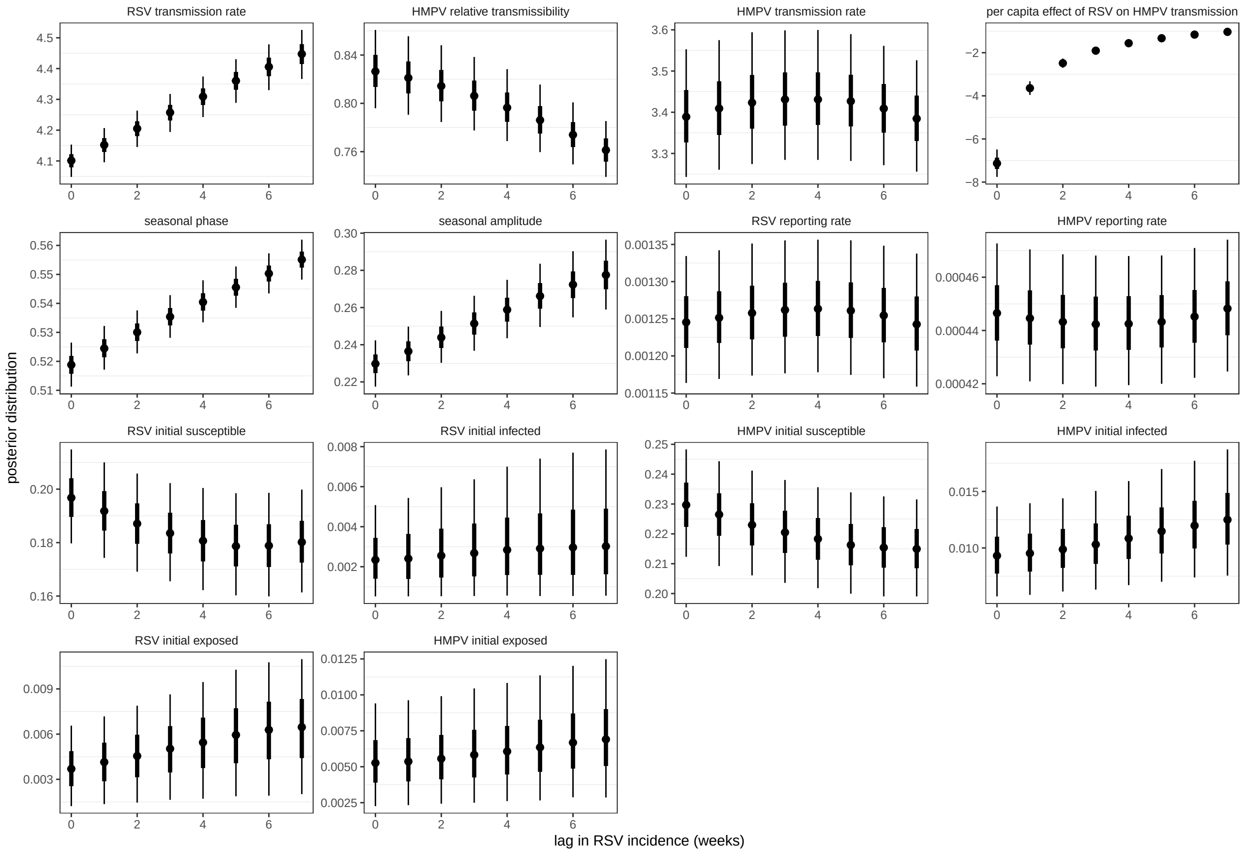

Supplementary Figure 11: Parameter estimates from fits of model allowing RSV incidence summed across prior weeks to affect current hMPV transmission rate. The model was refit using lags of 0 weeks (i.e., current RSV incidence only) to 7 weeks (i.e., prior 7 weeks plus current week summed). Circle shows median estimate from posterior distribution of $n = 7500$, thick lines show 50% credible interval, and thin lines show 90% credible interval.

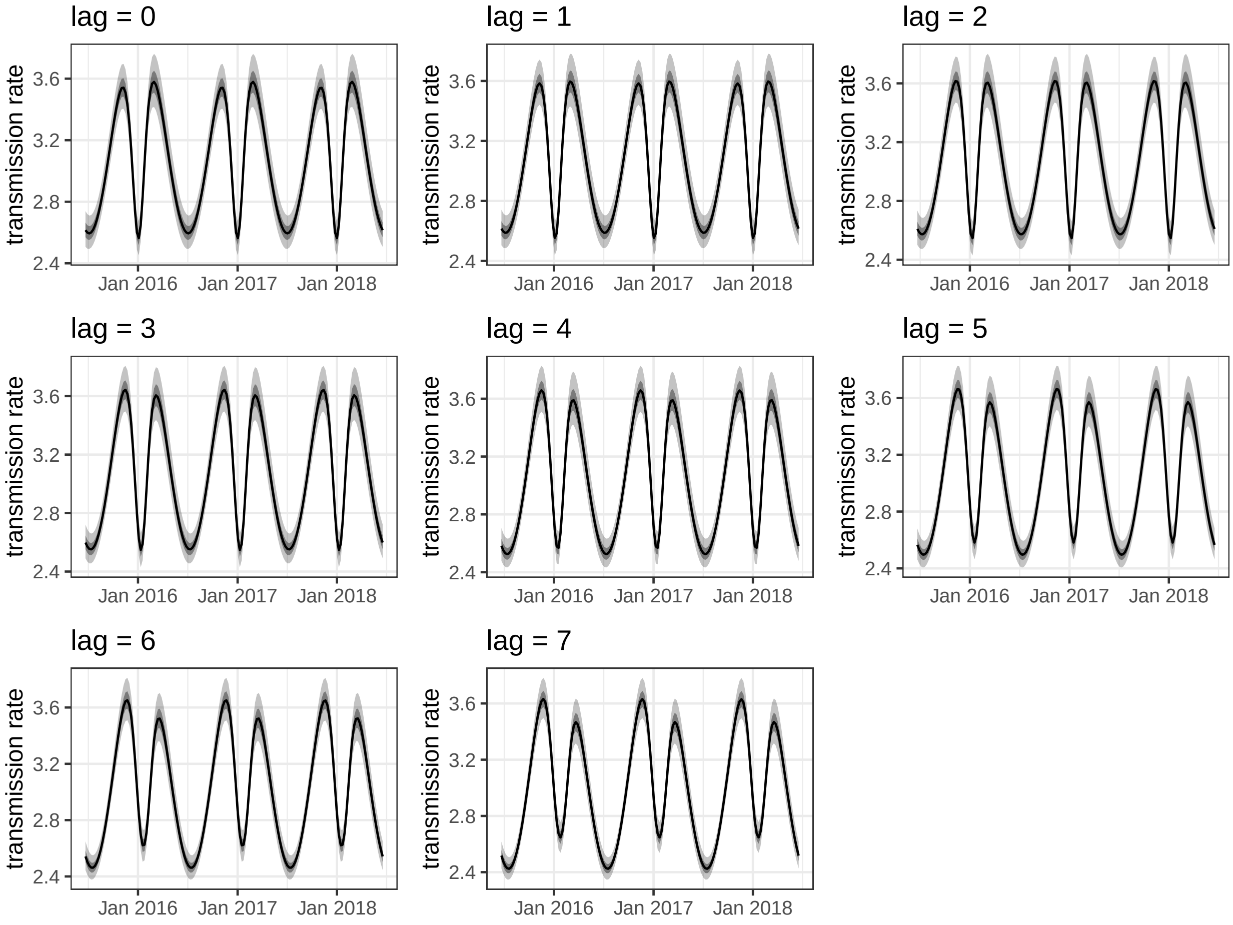

Supplementary Figure 12: Seasonal transmission rate for hMPV from fits of model allowing RSV incidence summed across prior weeks to affect current hMPV transmission rate. The model was refit using lags of 0 weeks (i.e., current RSV incidence only) to 7 weeks (i.e., prior 7 weeks plus current week summed). Lines show median estimate from the posterior distribution of $n = 7500$, dark ribbons show 50% credible interval, and light ribbons show 90% credible interval. Seasonal transmission rate is plotted for a subset of years, although the general shape of the seasonal curve holds throughout the fitting period.

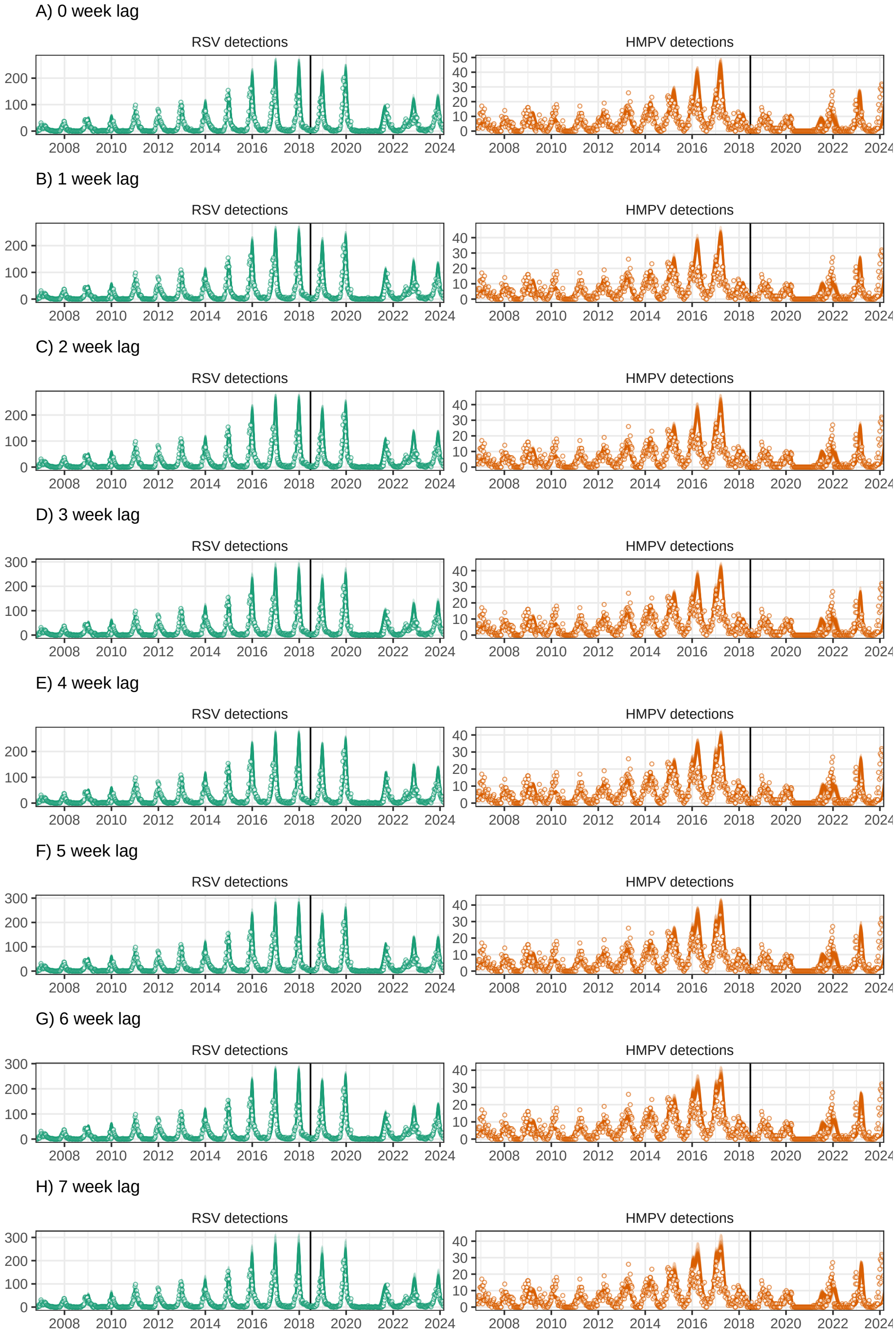

Supplementary Figure 13: Model fits and post-pandemic simulations using model that allows RSV incidence summed across prior weeks to affect current hMPV transmission rate. Fits are shown for models that use lags of 0 weeks (i.e., current RSV incidence only) to 7 weeks (i.e., prior 7 weeks plus current RSV incidence summed). Lines show 100 randomly selected model simulations for RSV (green) and hMPV (orange), open circles show observations, and black horizontal line shows date on which fitting period ends and out-of-sample simulations begin.

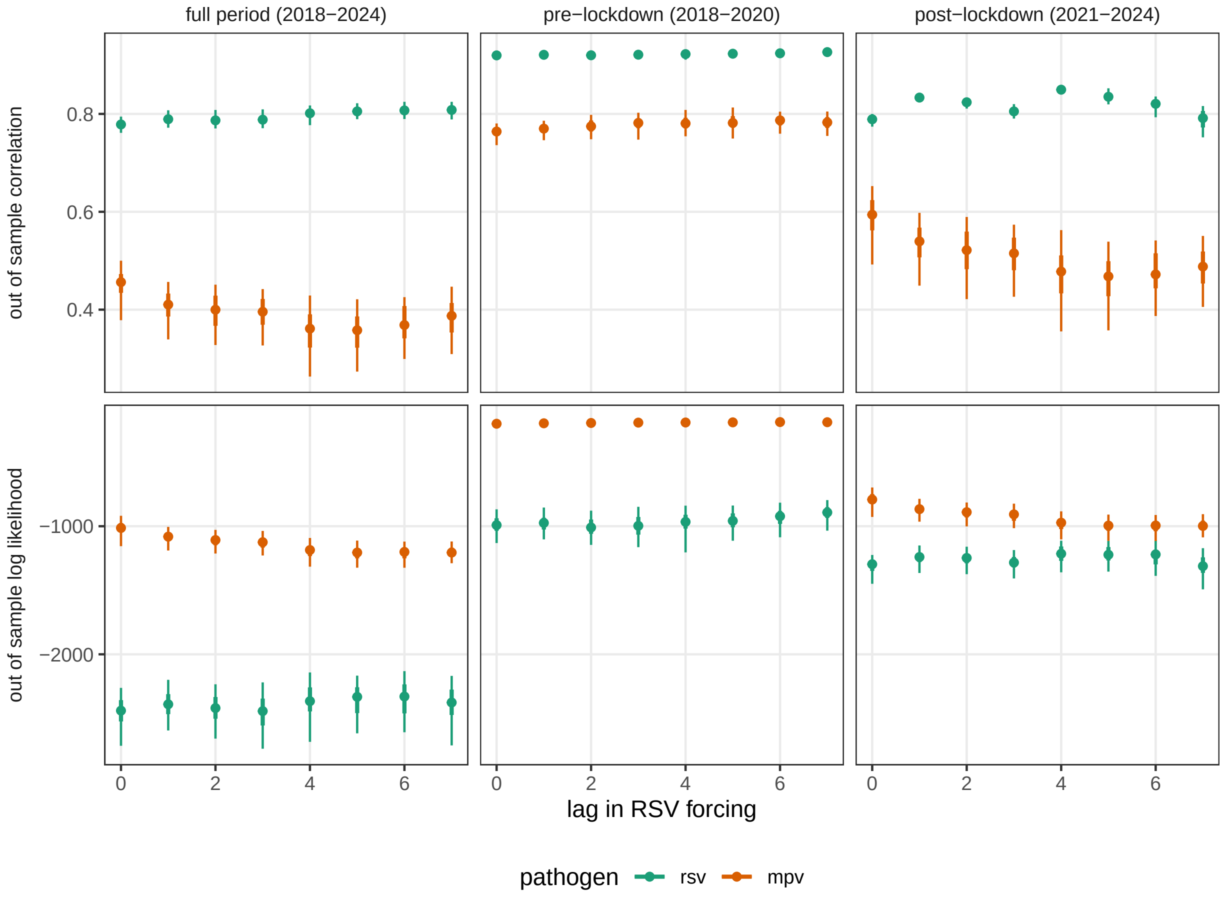

Supplementary Figure 14: Out of sample performance using model that allows RSV incidence summed across prior weeks to affect current hMPV transmission rate. Performance is shown for models that use lags of 0 weeks (i.e., current RSV incidence only) to 7 weeks (i.e., prior 7 weeks plus current RSV incidence summed). The distribution of performance across $n = 7500$ posterior samples for RSV (green) and hMPV (orange) is summarized by a Q5-Q95 (thin line), Q25-Q75 (thick line), and Q50 (circle). See Methods and Figure 4 caption for details.

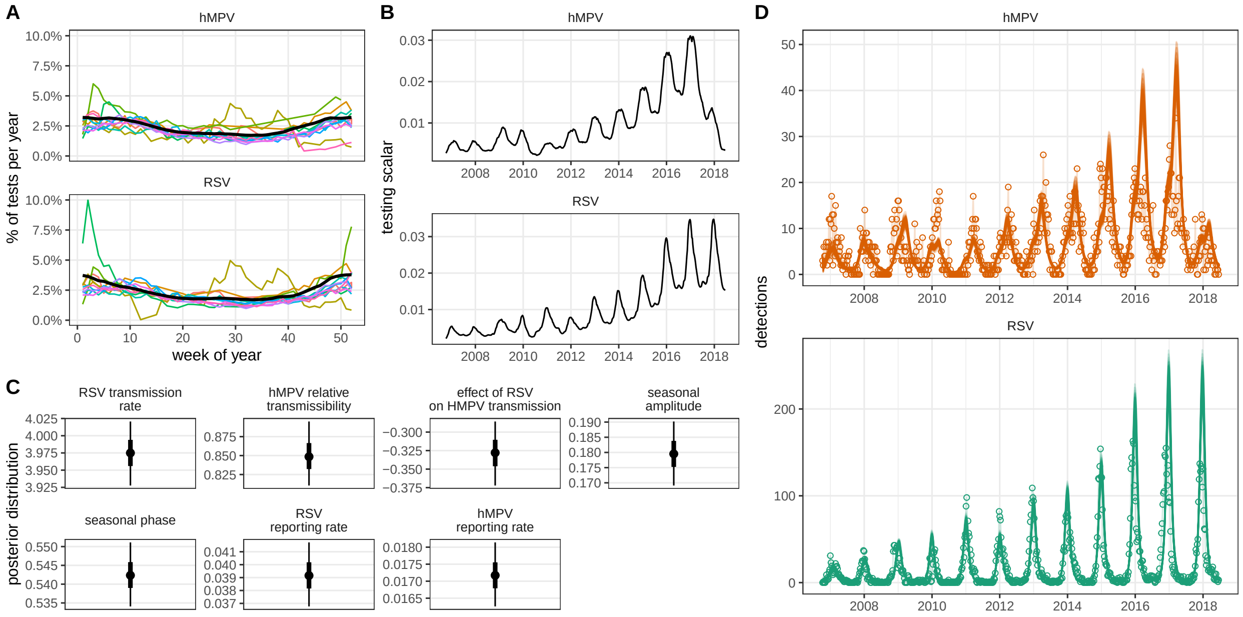

Supplementary Figure 15: Sensitivity of results to assumptions about testing. Here we consider weekly trends in testing in addition to long-term trends (as captured through the 1-year rolling average in testing in Supplementary Figure 3). (A) Percent of tests performed in each week across years between 2006 and 2018 (colored lines). An 8 week rolling average is calculated (black line). (B) Testing scalar that incorporates weekly testing trends used in model fitting. (C) Fitted parameters when weekly testing trends are incorporated. Circle shows median estimate from the posterior distribution of $n = 7500$, thick lines show 50% credible interval, and thin lines show 90% credible interval.(D) 100 posterior simulations from the fitted model (lines) and observations (points) for RSV (green) and hMPV (orange).

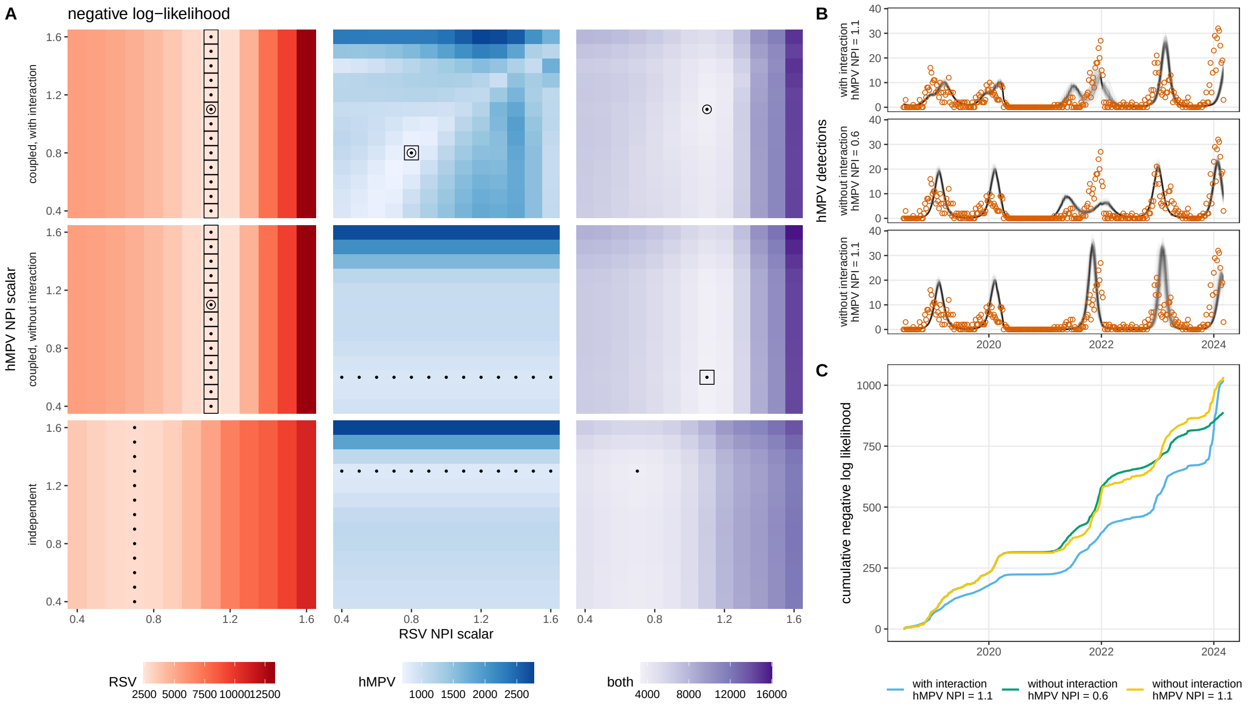

Supplementary Figure 16: Sensitivity of post-pandemic performance results to assumptions about NPI intensity. (A) Negative log likelihood (NLL) of post-pandemic simulations for RSV (red, first column), hMPV (blue, second column) and combined (purple, third column) under different NPI scalars for RSV (x-axis) and hMPV (y-axis). NPI scalars are used to transform Google mobility data into a change in the transmission rate (see Methods for details). In the main text, we assumed that NPIs reduced transmission via the same scalar for RSV and hMPV (i.e., along the diagonal), and here we relax that assumption. We tested the performance for three different models (rows), including the coupled (top row) and independent (bottom row) models from the main text. We also compared results to a third model (middle row) where the interaction term in the coupled model is removed (i.e., $c = 0$). Points show the scalar combination that minimizes NLL for each model, open circles show the combination that minimizes NLL across models assuming the RSV and hMPV scalars are equal (as in the main text), and the open squares show the combination that minimizes NLL across models when relaxing the assumption of equal scalars. In cases where there is no effect of one pathogen on the other (all RSV results, and models without interaction for hMPV), the NLL is independent of the NPI scalar for the other pathogen (hence multiple optimal values). (B) Dynamics of hMPV for the coupled model with interaction and without interaction for NPI scalars that optimize NLL across both pathogens (RSV NPI scalar = 1.1, hMPV NPI scalar = 0.6 or 1.1 depending on assumptions about equivalent scalars). We do not show the independent model as it never provided optimal NLL. When the NPI scalar is 0.6, the model without interaction does not capture the qualitative dynamics of the first rebound outbreak but captures the second two rebounds well. When the NPI scalar is 1.1, the model without interaction captures the timing and magnitude of the rebound outbreaks well. However, this can be attributed to the model overestimating the 2019-2020 season which depletes the pool of susceptible individuals and enables a larger, later initial outbreak. (C) Cumulative NLL over out of sample period for each model shown in panel B.

References

1. He, D., Ionides, E. L. & King, A. A. Plug-and-play inference for disease dynamics: measles in large and small populations as a case study. *Journal of The Royal Society Interface* **7,** 271–283. https://royalsocietypublishing.org/doi/10.1098/rsif.2009.0151 (2024) (June 2009).
2. Bjørnstad, O. N. *Epidemics: Models and Data using R* en. ISBN: 978-3-319-97486-6. https://www.springer.com/gp/book/9783319974866 (2021) (Springer International Publishing, 2018).
3. Mossong, J. *et al.* Social Contacts and Mixing Patterns Relevant to the Spread of Infectious Diseases. en. *PLOS Medicine* **5,** e74. https://journals.plos.org/plosmedicine/article?id=10.1371/journal.pmed.0050074 (2020) (Mar. 2008).
4. Reis, J. & Shaman, J. Retrospective Parameter Estimation and Forecast of Respiratory Syncytial Virus in the United States. en. *PLOS Computational Biology* **12,** e1005133. https://journals.plos.org/ploscompbiol/article?id=10.1371/journal.pcbi.1005133 (2024) (Oct. 2016).
5. White, L. J., Waris, M., Cane, P. A., Nokes, D. J. & Medley, G. F. The transmission dynamics of groups A and B human respiratory syncytial virus (hRSV) in England and Wales and Finland: seasonality and cross-protection. *Epidemiology and Infection* **133,** 279–289. https://www.ncbi.nlm.nih.gov/pmc/articles/PMC2870247/ (Apr. 2005).
6. Of Scotland, N. R. *2022 Census Geography Products* https://www.nrscotland.gov.uk/publications/2022-census-geography-products/. 2024.
7. National Records of Scotland. *National Records of Scotland* Document. May 2013. https://www.nrscotland.gov.uk/statistics-and-data/statistics/statistics-by-theme/vital-events/general-publications/weekly-births-registered-in-scotland (2024).
8. Nakajo, K. & Nishiura, H. Age-Dependent Risk of Respiratory Syncytial Virus Infection: A Systematic Review and Hazard Modeling From Serological Data. eng. *The Journal of Infectious Diseases* **228,** 1400–1409. ISSN: 1537-6613 (Nov. 2023).
