# Supplementary material for: Using COVID-19 pandemic perturbation to model RSV-hMPV interactions and potential implications under RSV interventions
Source: Nat Commun. 2025 Aug 6;16:7261. doi: 10.1038/s41467-025-62358-w (PMC12328645; doi:10.1038/s41467-025-62358-w)
Supplement: Supplementary file 3 — Reporting Summary [file 41467_2025_62358_MOESM3_ESM.pdf]

nature portfolio

Corresponding author(s): E. Howerton

Last updated by author(s): 06/30/2025

Reporting Summary

Nature Portfolio wishes to improve the reproducibility of the work that we publish. This form provides structure for consistency and transparency in reporting. For further information on Nature Portfolio policies, see our Editorial Policies and the Editorial Policy Checklist.

Statistics

For all statistical analyses, confirm that the following items are present in the figure legend, table legend, main text, or Methods section.

n/a	Confirmed	
☐	☒	The exact sample size (*n*) for each experimental group/condition, given as a discrete number and unit of measurement
☒	☐	A statement on whether measurements were taken from distinct samples or whether the same sample was measured repeatedly
☐	☒	The statistical test(s) used AND whether they are one- or two-sided<br>*Only common tests should be described solely by name; describe more complex techniques in the Methods section.*
☐	☒	A description of all covariates tested
☐	☒	A description of any assumptions or corrections, such as tests of normality and adjustment for multiple comparisons
☐	☒	A full description of the statistical parameters including central tendency (e.g. means) or other basic estimates (e.g. regression coefficient) AND variation (e.g. standard deviation) or associated estimates of uncertainty (e.g. confidence intervals)
☒	☐	For null hypothesis testing, the test statistic (e.g. *F*, *t*, *r*) with confidence intervals, effect sizes, degrees of freedom and *P* value noted<br>*Give P values as exact values whenever suitable.*
☐	☒	For Bayesian analysis, information on the choice of priors and Markov chain Monte Carlo settings
☒	☐	For hierarchical and complex designs, identification of the appropriate level for tests and full reporting of outcomes
☒	☐	Estimates of effect sizes (e.g. Cohen's *d*, Pearson's *r*), indicating how they were calculated

Our web collection on statistics for biologists contains articles on many of the points above.

Software and code

Policy information about availability of computer code

Data collection	Data from Canada was scraped from publicly available websites (information in Data Availability Statement) using the xml2 and rvest pacakges in R. All other data was collected manually. Scraping code can be found in a GitHub repository available at https://github.com/eahowerton/hmpv-rsv-intervention and archived at https://doi.org/10.5281/zenodo.15212115. For a complete list of packages used and corresponding versions, see the renv.lock file in this repository.
Data analysis	All code used to perform this analysis can be found in a GitHub repository available at https://github.com/eahowerton/hmpv-rsv-intervention and archived at https://doi.org/10.5281/zenodo.15212115. For a complete list of packages used and corresponding versions, see the renv.lock file in this repository.

For manuscripts utilizing custom algorithms or software that are central to the research but not yet described in published literature, software must be made available to editors and reviewers. We strongly encourage code deposition in a community repository (e.g. GitHub). See the Nature Portfolio guidelines for submitting code & software for further information.

Data

Policy information about availability of data

All manuscripts must include a data availability statement. This statement should provide the following information, where applicable:

- Accession codes, unique identifiers, or web links for publicly available datasets
- A description of any restrictions on data availability
- For clinical datasets or third party data, please ensure that the statement adheres to our policy

The RSV and hMPV outbreak data from Scotland are available upon request to NHSGGC/NHS Scotland (https://www.informationgovernance.scot.nhs.uk/pbpphsc/home/for-applicants/). Data on weekly incidence of RSV and hMPV for Canada and Korea were collected from publicly available sources. Data for Canada were scraped from the historical Respiratory Virus Detection Surveillance System reports provided by Public Health Agency of Canada, available at https://www.canada.ca/en/public-health/services/surveillance/respiratory-virus-detections-canada.html. Data for Korea was downloaded from the Acute Respiratory Infection section of the Korea Disease Control and Prevention Agency Infectious Disease Statistics website, available at https://dportal.kdca.go.kr/pot/index.do. Demographic data for Scotland was downloaded from the Weekly Births in Scotland report provided by National Records of Scotland (https://www.nrscotland.gov.uk/statistics-and-data/births-deaths-marriages-and-life-expectancy/#). Google mobility data for the UK was downloaded from the Community Mobility Reports provided at https://www.google.com/covid19/mobility/. All data, including raw data and posteriors derived from model fitting, can be found in the /data folder of the GitHub repository available at https://github.com/eahowerton/hmpv-rsv-intervention and archived at {https://zenodo.org/records/15778459.

Research involving human participants, their data, or biological material

Policy information about studies with human participants or human data. See also policy information about sex, gender (identity/presentation), and sexual orientation and race, ethnicity and racism.

Reporting on sex and gender	Sex and gender information was not included in this study.
Reporting on race, ethnicity, or other socially relevant groupings	Race and ethnicity information was not included in this study.
Population characteristics	Patient-specific information including age, prior health history, etc. was not used in this study. Analyses were performed directly on summarized incidence data.
Recruitment	In Scotland, samples were collected from patients with respiratory illness in primary and secondary care settings, and thus the data is biased towards symptomatic infections.
Ethics oversight	Given all data are anonymized and most are publicly available, IRB considerations do not apply.

Note that full information on the approval of the study protocol must also be provided in the manuscript.

Field-specific reporting

Please select the one below that is the best fit for your research. If you are not sure, read the appropriate sections before making your selection.

☐ Life sciences ☐ Behavioural & social sciences ☒ Ecological, evolutionary & environmental sciences

For a reference copy of the document with all sections, see nature.com/documents/nr-reporting-summary-flat.pdf

Ecological, evolutionary & environmental sciences study design

All studies must disclose on these points even when the disclosure is negative.

Study description	This modeling study assesses population-level RSV and hMPV outbreak dynamics and estimates potential virus-virus interactions that could contribute to these patterns. Our primary analysis quantifies potential interactions from weekly RSV and hMPV incidence in Scotland. Thus, we focus our answers below on this data source.
Research sample	Data was provided by the West of Scotland Testing Centre. PCR testing results for RSV and hMPV were summarized into weekly incidence for each pathogen.
Sampling strategy	Samples were collected via convenience sample from patients with respiratory illness in primary and secondary care settings. Temporal changes in sample collection were accounted for in the model.
Data collection	The West of Scotland Specialist Testing Centre processed samples and recorded results.
Timing and spatial scale	This study analyzed RSV and hMPV incidence in Scotland from 2006-10-09 to 2024-03-04. Samples were primarily collected from the Glasgow area and the west of Scotland, although some national surveillance samples were also included each year.
Data exclusions	PCR tests that were equivocal or insufficient were excluded from weekly incidence.

Reproducibility	This modeling study can be fully reproduced using code provided in the above github repository. Scotland data cannot be made publicly available.
Randomization	This study aims to analyze historical patterns in reported RSV and hMPV incidence via a mechanistic transmission model. Thus, randomization is not relevant.
Blinding	This study aims to analyze historical patterns in reported RSV and hMPV incidence via a mechanistic transmission model. Thus, blinding is not relevant.

Did the study involve field work? ☐ Yes ☒ No

Reporting for specific materials, systems and methods

We require information from authors about some types of materials, experimental systems and methods used in many studies. Here, indicate whether each material, system or method listed is relevant to your study. If you are not sure if a list item applies to your research, read the appropriate section before selecting a response.

Materials & experimental systems

n/a	Involved in the study
☒	☐ Antibodies
☒	☐ Eukaryotic cell lines
☒	☐ Palaeontology and archaeology
☒	☐ Animals and other organisms
☒	☐ Clinical data
☒	☐ Dual use research of concern
☒	☐ Plants

Methods

n/a	Involved in the study
☒	☐ ChIP-seq
☒	☐ Flow cytometry
☒	☐ MRI-based neuroimaging

Plants

Seed stocks	*Report on the source of all seed stocks or other plant material used. If applicable, state the seed stock centre and catalogue number. If plant specimens were collected from the field, describe the collection location, date and sampling procedures.*
Novel plant genotypes	*Describe the methods by which all novel plant genotypes were produced. This includes those generated by transgenic approaches, gene editing, chemical/radiation-based mutagenesis and hybridization. For transgenic lines, describe the transformation method, the number of independent lines analyzed and the generation upon which experiments were performed. For gene-edited lines, describe the editor used, the endogenous sequence targeted for editing, the targeting guide RNA sequence (if applicable) and how the editor was applied.*
Authentication	*Describe any authentication procedures for each seed stock used or novel genotype generated. Describe any experiments used to assess the effect of a mutation and, where applicable, how potential secondary effects (e.g. second site T-DNA insertions, mosiacism, off-target gene editing) were examined.*
